# Operational strategies of anti-malarial drug campaigns for malaria elimination in Zambia’s southern province: a simulation study

**DOI:** 10.1186/s12936-016-1202-0

**Published:** 2016-03-09

**Authors:** Erin M. Stuckey, John M. Miller, Megan Littrell, Nakul Chitnis, Rick Steketee

**Affiliations:** Swiss Tropical and Public Health Institute, Basel, Switzerland; University of Basel, Basel, Switzerland; Bill & Melinda Gates Foundation, Seattle, WA USA; PATH MACEPA, Lusaka, Zambia; PATH MACEPA, Seattle, WA USA

**Keywords:** Malaria, Modelling, Elimination, *Plasmodium falciparum*, Primaquine, Ivermectin, Zambia

## Abstract

**Background:**

Malaria elimination requires reducing both the potential of mosquitoes to transmit parasites to humans and humans to transmit parasites to mosquitoes. To achieve this goal in Southern province, Zambia a mass test and treat (MTAT) campaign was conducted from 2011–2013 to complement high coverage of long-lasting insecticide-treated nets (LLIN). To identify factors likely to increase campaign effectiveness, a modelling approach was applied to investigate the simulated effect of alternative operational strategies for parasite clearance in southern province.

**Methods:**

OpenMalaria, a discrete-time, individual-based stochastic model of malaria, was parameterized for the study area to simulate anti-malarial drug administration for interruption of transmission. Simulations were run for scenarios with a range of artemisinin-combination therapies, proportion of the population reached by the campaign, targeted age groups, time between campaign rounds, *Plasmodium falciparum* test protocols, and the addition of drugs aimed at preventing onward transmission. A sensitivity analysis was conducted to assess uncertainty of simulation results. Scenarios were evaluated based on the reduction in all-age parasite prevalence during the peak transmission month one year following the campaign, compared to the currently-implemented strategy of MTAT 19 % population coverage at pilot and 40 % coverage during the first year of implementation in the presence of 56 % LLIN use and 18 % indoor residual spray coverage.

**Results:**

Simulation results suggest the most important determinant of success in reducing prevalence is the population coverage achieved in the campaign, which would require more than 1 year of campaign implementation for elimination. The inclusion of single low-dose primaquine, which acts as a gametocytocide, or ivermectin, which acts as an endectocide, to the drug regimen did not further reduce parasite prevalence one year following the campaign compared to the currently-implemented strategy. Simulation results indicate a high proportion of low-density infections were missed by rapid diagnostic tests that would be treated and cleared with mass drug administration (MDA).

**Conclusions:**

The optimal implementation strategy for MTAT or MDA will vary by background level of prevalence, by rate of infections imported to the area, and by ability to operationally achieve high population coverage. Overall success with new parasite clearance strategies depends on continued coverage of vector control interventions to ensure sustained gains in reduction of disease burden.

## Background

Malaria elimination requires reducing both the potential of mosquitoes to transmit parasites to humans and humans to transmit parasites to mosquitoes, including reaching humans with asymptomatic infections. With the backdrop of several years of high coverage of long-lasting insecticide-treated mosquito nets (LLINs), many countries are looking to reorient their malaria programmes from control to elimination, including the adoption of additional strategies for programme implementation and surveillance.

Zambia has a long history of malaria control efforts dating back to the 1920s with the mining sector in the copperbelt [1]. With the advent of the Roll Back Malaria initiative in 1998, Zambia began to introduce additional insecticide-treated bed nets (ITNs) as part of small-scale delivery programmes targeting vulnerable populations. Following initiation of malaria funding from the Global Fund for AIDS, Tuberculosis and Malaria and other partners, a scaling up of key malaria interventions, including mass distribution of ITNs, renewed efforts to increase IRS coverage, artemisinin-combination therapy (ACT) for anti-malarial treatment, and rapid diagnostic tests for case management began to reach communities by 2005. This scaling up for impact (SUFI) approach contributed to an observed 63 % decrease in *Plasmodium falciparum* prevalence in ages 2–9 and a 22 % decrease in malaria deaths [2]. In response to the need to plan next steps to accelerate the goal of malaria elimination the Zambia national malaria control centre (NMCC) in partnership with PATH MACEPA is conducting an evaluation of strategies for anti-malarial drug campaigns. These strategies are designed to complement the existing high coverage of vector control interventions and aim to contribute to a marked reduction of community level malaria. In addition to the 2011–2013 implementation strategy of mass test and treat (MTAT) targeting all ages with three rounds of artemether-lumefantrine (AL) [3] an additional set of operational and chemotherapeutic options are currently under evaluation [4]. These changes to the design include replacing AL with another ACT, dihydroartemisinin + piperaquine (DHAP), which clears asexual blood stages of current infections and has a longer prophylactic period against future malaria infection [5], as well as use of single low-dose (SLD) primaquine [6] and/or ivermectin [7] to stop onward transmission to humans.

Primaquine is a gametocytocidal drug that reduces the duration of late-stage *P. falciparum* gametocytes, the transmissible stage of the parasite from human to mosquito, and therefore has the potential to reduce malaria transmission [8]. The World Health Organization (WHO) currently recommends the use of SLD primaquine in addition to an ACT for all patients with parasitologically-confirmed *P. falciparum* malaria to aid in malaria elimination and to contain the spread of artemisinin resistance [9]. However, this use of primaquine is not currently widespread due to the limited availability of appropriate dosing regimens for young children and some residual confusion regarding the safety of SLD versus standard higher dosing of primaquine in patients with glucose-6-phosphate dehydrogenase (G6PD) deficiency. Clinical trials to determine the lowest possible dose effective at clearing gametocytes are in process in a number of locations considering the inclusion of the drug in elimination strategies including Uganda [10], The Gambia [11], and Mali [12].

Ivermectin is an anthelmintic drug currently used for control of onchocerciasis and lymphatic filariasis. It also has endectocidal properties reducing the sporogony [13] and survivorship [14] of mosquitoes that take a blood meal from humans with the drug in their system. Studies indicate that mass drug administration (MDA) with ivermectin has the potential to assist in the interruption of malaria transmission [15, 16], and has the potential to combine malaria elimination efforts with other helminth-based diseases considered for elimination.

OpenMalaria is a stochastic individual-based model of malaria that has been used to address questions relevant to malaria epidemiology, vaccine development [17], cost-effectiveness of different combinations of malaria control interventions [18], and vector control technologies [19]. In order to assist the National Malaria Control Programme and implementing partners in Zambia with model-based guidance on community-based treatment strategies, OpenMalaria was employed to simulate the effectiveness of changing the operational strategy of the malaria testing and treatment campaign. This paper presents the simulation results of MTAT with AL, MTAT with DHAP (with or without ivermectin), and MDA with DHAP (with or without ivermectin and/or SLD primaquine).

## Methods

### OpenMalaria model

Alternative strategies and drug interventions against malaria parasites and the mosquito vector were evaluated using OpenMalaria. OpenMalaria combines stochastic individual-based simulation models for malaria in humans with a periodically-forced deterministic model for malaria in mosquitoes [20–23]. The models have been fitted to multiple field data sets and the models for malaria in humans include demography, acquired immunity and super-infection, variations in parasite densities and infectiousness to mosquitoes and the clinical effects of malaria. The model for malaria in mosquitoes includes multiple mosquito species, varying vector behaviour and non-human hosts [20–23]. The model platform consists of an ensemble of fourteen model variants that allow different assumptions such as heterogeneity in exposure and decay of immunity to help quantify the effects of uncertainty in model formulation [23]. Details of the model and simulation set up can be found in the references above.

### Baseline scenario parameterization

**Fig. 1:**
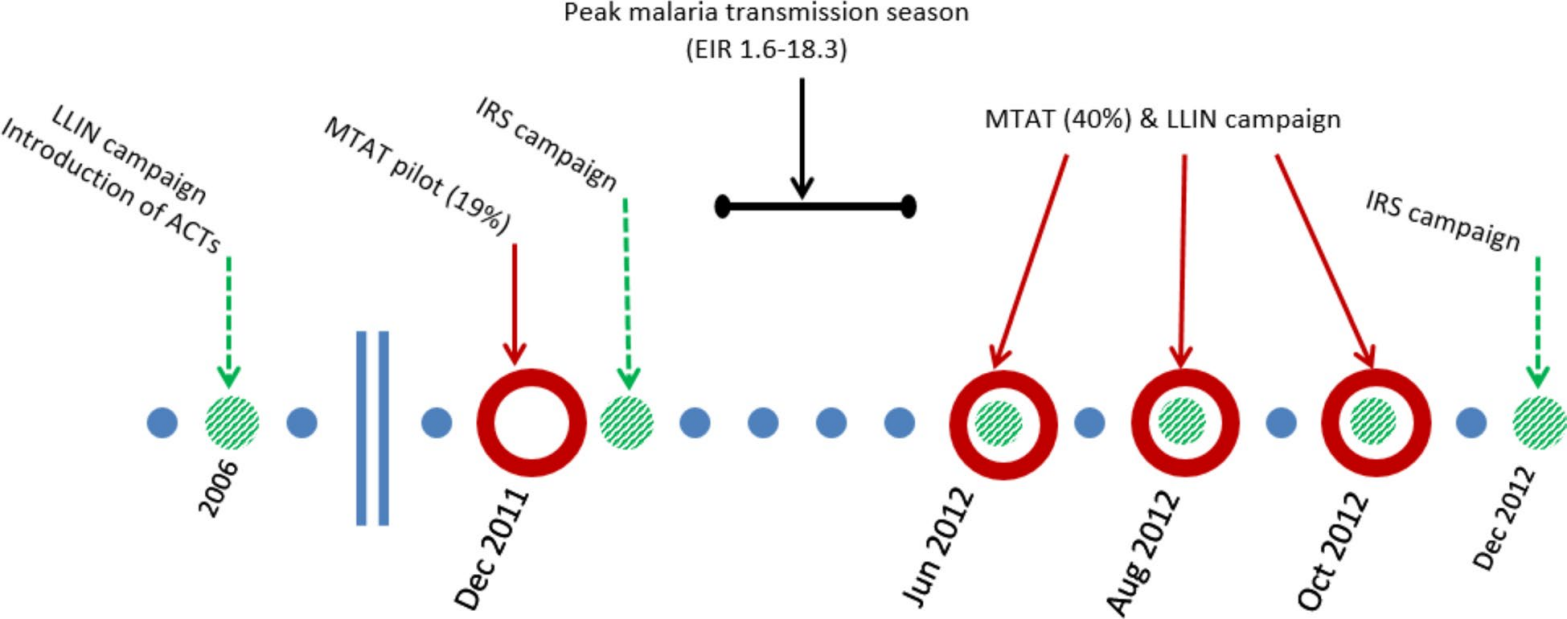
Schematic of malaria control interventions existing in Southern province, Zambia from 2006 to 2012 that served to inform the baseline scenario used for this experiment. The *blue circles* correspond to months with no active deployment of malaria control interventions. The *green circles* corresponds to months where vector control interventions were deployed and the *open red circles* correspond to months were drug interventions were deployed

The baseline scenario parameterization incorporated published literature and available data from field studies to represent the context of malaria in Zambia’s Southern province, where elimination strategies in the country were initiated. The seasonal pattern of malaria transmission corresponds to the majority of infections occurring from January to May with *Anopheles gambiae* and *A. funestus* as the primary vectors [24, 25]. The baseline case management system assumes 21.8 % of fever cases access an anti-malarial, either the recommended first line anti-malarial drug (AL) introduced in 2006 [26] or sulfadoxine–pyrimethamine (SP), the second most commonly-used anti-malarial [25]. 81 % of the population is assumed to fully adhere to AL [27], and 39.4 % to adhere to SP [28]. Non-adherers to AL receive a reduced probability of clearing the infection, while all non-adherers to SP are assumed to not clear infections. Existing malaria control interventions described in Fig. 1. Coverage of any given intervention is assumed to be independent of coverage of any other intervention. Simulations assume 71.8 % of the population in the study area own an LLIN, 41.3 % of the population slept under a net the previous night [29], and 18 % of households received indoor residual spraying (IRS). Coverage of MTAT interventions were chosen based on the preliminary results from the MTAT pilot in 2011 and 2012. Overall, 19 % of the simulated population received an MTAT intervention in December 2011; in each of the 2012 dry season rounds, 40 % of the simulated individuals received an MTAT intervention so that over three rounds approximately 78 % of the simulated population received at least one malaria test.

This baseline scenario was simulated on a population of 10,000 individuals with Zambia’s population age structure [30]. Simulation results from the baseline scenario were validated against observed rapid diagnostic test (RDT) positivity rates by facility catchment area following the scale up of the MTAT programme using AL [29]. Results were stratified by baseline parasite prevalence, taking into account variations in transmission intensity.

### Experiment design and analysis

To investigate the effects of operational considerations of MTAT with AL on health outcomes, assuming the baseline MTAT implementation as illustrated in Fig. 1, the scenarios described in Table 1 were included in the experiment and altered one at a time. These include varying the age groups targeted by the campaign, varying the time between campaign delivery rounds, and varying the proportion of the population reached by the campaign. MTAT campaign coverage numbers were chosen to correspond to the preliminary findings of coverage achieved by the 2011–2012 intervention implementation. Age groups were chosen to correspond to the groups with the highest parasite prevalence rates. To investigate the effects of changing the drug regimen on health outcomes of the MTAT intervention, scenarios were simulated with the longer-acting DHAP, both alone and paired with ivermectin (Table 2). To investigate the effects of removing the RDT and implementing an MDA campaign, scenarios were simulated with DHAP alone, DHAP + SLD primaquine, DHAP + ivermectin, and the combination of all three drugs (Table 2).

**Table 1:** Experiment design for mass test and treat (MTAT) with artemether-lumefantrine (AL)

Variable	Simulation levels
Target ages receiving the campaign	*All ages*, children under 5, children 5–14, adults 25–49
Time between MTAT delivery rounds (total of three rounds)	2, 3, 4, *8* weeks
Proportion of the population reached per round by the MTAT campaign	0, 40, *72*, 100 %
Proportion of the population who fully adhere to the AL drug regimen	50, *75*, 100 %

Baseline values indicated in italics

**Table 2:** Experiment design for campaign implementation with dihydroartemisinin–piperaquine (DHAP)

Drug combination	Population reached by MTAT campaign	Population reached by MDA campaign	Ivermectin duration of effectiveness	Single low-dose (SLD) primaquine half-life
Campaign implementation with DHAP	40, *72* %	40, *72* %		
DHAP + ivermectin	40, *72* %^a^	40, *72* %	*2*, 4, 7 days	
DHAP + SLD primaquine		40, *72* %		12, *48*, 72 h
DHAP + ivermectin + SLD primaquine		40, *72* %		

Baseline values indicated in italics

^a^In this scenario ivermectin is given to every individual reached by the campaign, regardless of the result of their malaria test

A sensitivity analysis was conducted to investigate the sensitivity of the intervention to changes in factors likely to influence the effect of the specific intervention or strategy. This includes the proportion of the population who fully adhere to the AL drug regimen (Table 1), and the duration of the effects of ivermectin and SLD primaquine (Table 2). In addition, each of the scenarios described above were simulated over fourteen model variants with varied assumptions about malaria epidemiology to account for model uncertainty [23], and three random seeds to account for the effects of stochasticity.

Intervention scenarios were evaluated based on the simulated reduction in mean all-age parasite prevalence taken over the 14 model variants and three random seeds (mean parasite prevalence) in the peak month of malaria transmission one year following the intervention compared to the baseline scenario.

### Intervention assumptions

#### Drugs

**Fig. 2:**
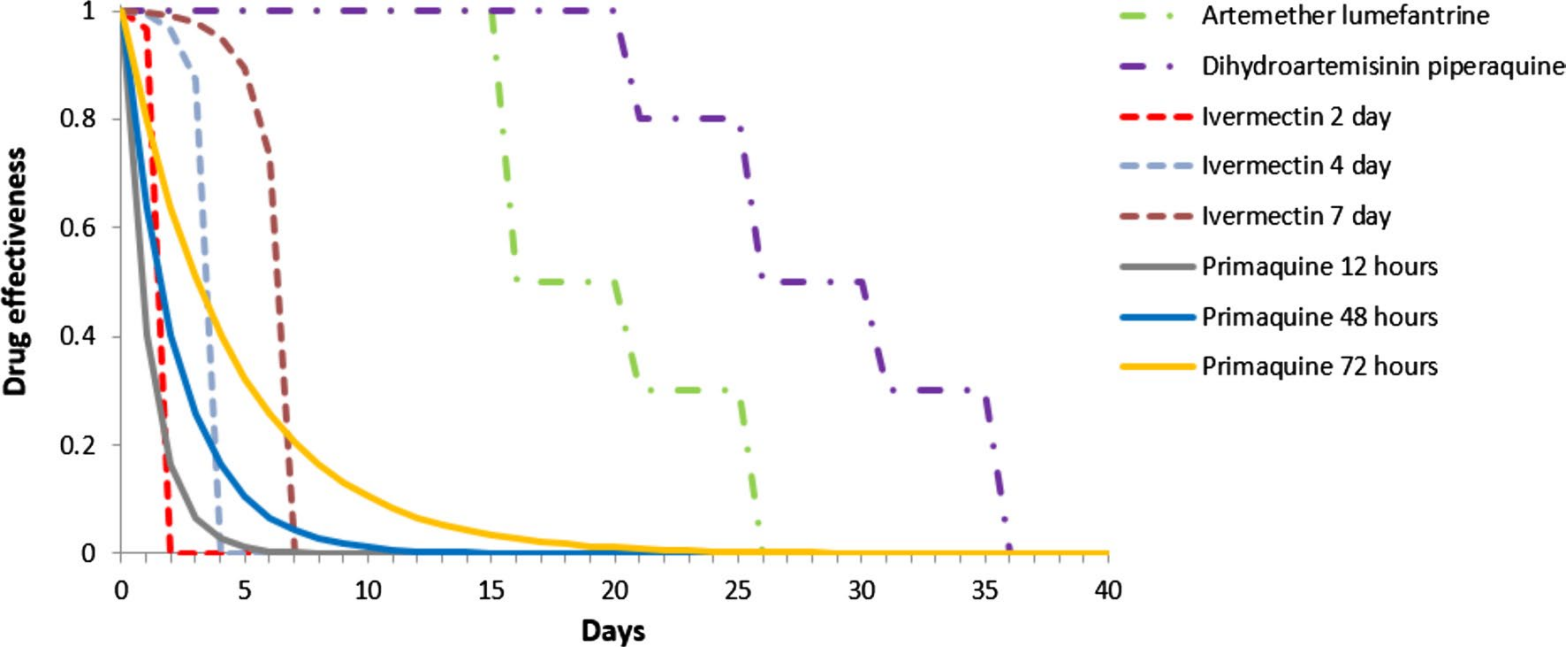
Assumptions about the decay in drug effectiveness. ACT (*dashed/dotted lines*) decay in a step-wise fashion with AL (*green*) lasting 25 days and DHAP (*purple*) 35 days. PQ (*solid line*) has an exponential decay with varying half-life of effectiveness of 12 (*gray*), 48 (*blue*), and 72 h (*orange*). Ivermectin (*dashed lines*) smoothly decays to efficacy of zero after 2 (*red*), 4 (*grey*), and 7 days (*brown*)

AL is assumed to clear 100 % of asexual blood stage infections [31] in individuals with full adherence to the drug regimen with a prophylactic period of 25 days [32]. DHAP is assumed to have an equivalent effect on clearing existing asexual blood stage infections as AL, but goes on to have a prophylaxis benefit for an additional 10 days [32] during which new infective mosquito bites do not go on to transmit infection. Ivermectin is given as a single dose with an 85 % killing effect on mosquitoes; killing time is varied to be either 2, 4 or 7 days according to the dose. Primaquine is given as a single low dose formulation and is assumed to immediately kill all sexual stage 5 parasites with a half-life of 2 days. However, all remaining earlier stage parasites and those arising from an incompletely cleared infection become stage 5 parasites and are able to be transmitted. Adherence rate for both AL and DHAP is assumed to be 75 % in the baseline simulations but is varied in the sensitivity analysis as described above. Because missing the final dose has been reported to be the most common reason for non-adherence [33] leading to a lower mean plasma lumefantrine level in individuals treated with AL [34], the probability of infection clearance is reduced by 15 % for non-adherers. Adherence to SLD primaquine and ivermectin is assumed to be 100 % given their administration at the start of the drug regimen and therefore likelihood of direct observation. Assumptions about the decay of effectiveness of the range of drugs simulated in this experiment are summarized in Fig. 2.

#### Delivery method

With MTAT implementation, an ACT is only given to individuals with a positive RDT result; thus, the sensitivity and specificity of the RDT establish the likely proportion of which individuals will test positive according to parasite density of the infection, and which individuals will be infected but test negative. These will typically be individuals with lower parasite density who may also have a lower likelihood of transmitting to mosquitoes. In scenarios including ivermectin with MTAT, while ACT is only given to individuals who test positive, ivermectin is given to every individual reached by the campaign, regardless of the result of their malaria test. This is because ivermectin is not a therapeutic drug for malaria treatment, but rather acts as an endectocide that is toxic to mosquitoes that bite an individual with the drug in their system. With MDA implementation, the drugs are given to everyone. However, this is moderated by the population coverage of the intervention; some people are reached and others are not, and an individual has the same independent likelihood of receiving the intervention in each delivery round.

#### Vector control

LLIN deterrency and pre- and post-prandial killing of malaria vector mosquitoes were parameterized using experimental hut data, as described in Briët et al. [19]. LLINs are assumed to decay over time at different rates for their physical state, chemical state, and attrition from circulation. IRS deterrency and post-prandial killing are assumed to decay over time with an exponential function and a half-life of 4 months. Both LLINs and IRS are assumed to only affect the proportion of indoor biting and resting mosquitoes. Additional details of the model description and parameterization of vector control interventions are described elsewhere [19, 35].

## Results

### MTAT with AL strategy used in 2011–2013

**Fig. 3:**
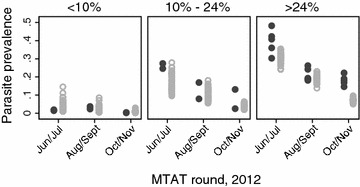
Validation of the baseline scenario parameterization: simulated versus observed effect of three rounds of MTAT using AL, by parasite prevalence at pilot conducted in December 2011 [29]. *Black circles* represent observed all-age mean parasite prevalence rates in health facility catchment areas. *Grey circles* represent simulated mean parasite prevalence rates

The model was able to simulate the range of observed prevalence of health facility catchment areas at each of the three MTAT deployment rounds in each of the three parasite prevalence strata at the time of the December 2011 pilot survey (Fig. 3). Both the observed and simulated data show the variance in prevalence between health facility catchment areas decreasing through the season. However, for the health facility catchment areas representing the highest parasite prevalence rate, the simulation runs under-estimated the peak parasite prevalence rate (no estimates were greater than 35 %) for the June/July intervention round when the observed prevalence rates reached as high as 48 % (Fig. 3) [28]. This trend was also observed in subsequent intervention rounds (Fig. 3).

**Fig. 4:**
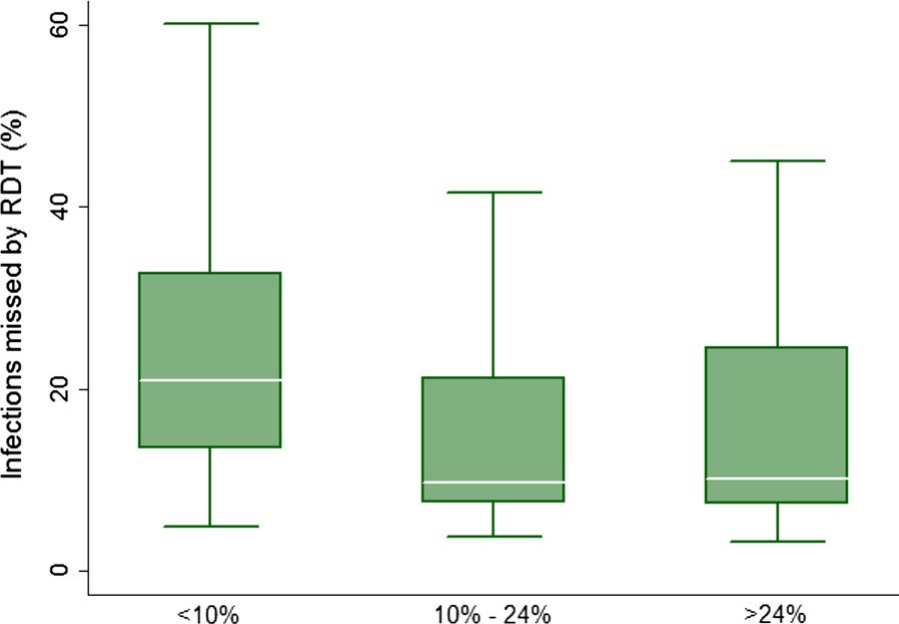
Prevalence of subpatent infections. The percentage of simulated infections in the population with parasitaemia of fewer than 100 parasites per microlitre, the simulated detection limit of the RDT used in the MTAT campaign. *White lines* represent the simulated median value, *green boxes* represent the inter-quartile range, and *capped bars* represent the* upper* and* lower* adjacent values for simulated results using an ensemble of 14 model variants and three random seeds for populations in each category of mean parasite prevalence

Results suggest a substantial proportion of infections in the population exist with parasite densities of fewer than 100 parasites per microlitre, the RDT detection limit assumed by the model. Simulations indicate the median proportion of infections that are sub patent is 20 % in scenarios with a baseline of less than 10 % mean parasite prevalence at the 2011 survey, dropping to 10 % in scenarios with baseline mean parasite prevalence in the two higher prevalence categories (Fig. 4).

### Changing operational strategy of MTAT campaign

**Fig. 5:**
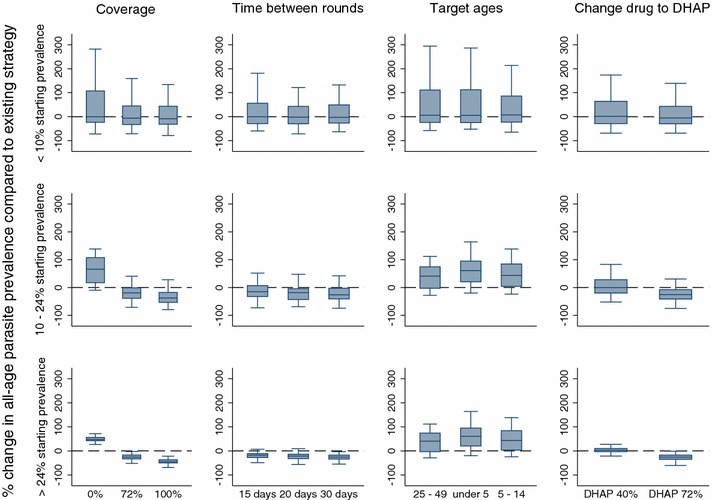
Results of changing operational strategy for mass test and treat using artemether-lumefantrine. Change (%) in the simulated mean parasite prevalence compared to the simulated mean parasite prevalence measured 1 year after implementation of the existing intervention strategy, as a result of varying the operational strategy of the intervention. Categories represented in the *columns* include the proportion of the population reached by the campaign (coverage), the number of days between screen and treat rounds (time between rounds), the ages reached by the intervention (target ages), and varying the anti-malarial drug used in the campaign (change drug to DHAP). The existing strategy consists of three rounds of mass test and treat with AL covering 40 % per round of all ages in the population with 60 days between treatment rounds. The *rows* represent results stratified by the pre-intervention mean parasite prevalence in the population as measured by RDT. *Centre solid lines* represent the simulated median value, *blue boxes* represent the inter-quartile range, and *capped bars* represent the* upper* and* lower* adjacent values for simulated results using an ensemble of 14 model variants and three random seeds

Changes in the proportion of the population reached by the campaign, the number of days between screen and treat rounds, and varying the anti-malarial drug used in the campaign had the greatest effect in areas with a starting mean parasite prevalence of greater than 24 %, and the least effect in areas with a starting mean parasite prevalence of less than 10 % (Fig. 5). A contributing factor to this effect could be the greater variation in simulated results in this lowest-prevalence category. For example, when simulating the parasite prevalence given 72 % coverage of MTAT with AL, the standard deviation for the <10 versus >24 % prevalence groups were ±62.5 and ±13.8, respectively (Table 3; Fig. 5).

**Table 3:** Effect of changing operational strategy compared to a baseline strategy of three rounds mass test and treat with artemether-lumefantrine at 40 % coverage per round of all ages of the population with 60 days between rounds

% Change in median parasite prevalence (IQR), by pre-intervention mean parasite prevalence											
%	Coverage (%)			Frequency (days)			Ages			Change drug (%)	
	0	72	100	15	20	30	Under 5	5–14	25–49	DHAP 40	DHAP 72
<10	0.0 (−23.7, 107.4)	−6.1 (−32.6, 44.7)	−8.7 (−31.6, 43.4)	0 (−28.9, 56.3)	−2.4 (−30.0, 42.6)	−2.9 (−26.3, 48.9)	5.5 (−24.7, 112.6)	7.4 (−23.2, 86.1)	5.8 (−23.9, 111.3)	1.6 (−28.9, 64.2)	−5.5 (−30.0, 43.2)
10–24	65.8 (17.5, 107.1)	−*19.7* (−*38.8,* −*1.0*)	−*37.6* (−*53.2,* −*17.8*)	−15.1 (−32.5, 6.7)	−*18.6* (−*43.2,* −*4.4*)	−*26.2* (−*40.8,* −*2.8*)	60.8 (20.6, 95.2)	43.9 (4.1, 84.4)	40.9 (−2.9, 74.3)	−0.8 (−20.1, 28.1)	−*25.5* (−*41.4,* −*7.4*)
>24	47.5 (42.7, 56.3)	−*25.6* (−*33.1,* −*16.8*)	−*44.2* (−*49.9,* −*36.6*)	−*18.2* (−*27.5,* −*11.1*)	−*21.4* (−*31.8,* −*13.6*)	−*25.3* (−*33.3,* −*17.2*)	46.3 (37.2, 51.34)	36.8 (29.1, 49.7)	29.8 (19.4, 38.1)	2.9 (−3.1, 9.8)	−*25.7* (−*34.5,* −*17.2*)

Italics indicate results that perform better than the baseline strategy

Targeting specific age groups for the MTAT intervention, as opposed to reaching all-ages as in the current MTAT strategy, resulted in an increase in prevalence (reduced efficacy) in all prevalence categories. This is because a smaller proportion of the population is reached, even though this proportion of the population may have a higher mean parasite prevalence. Unlike other operational strategy changes, changing the time between campaign rounds to 30 days or less showed a greater simulated benefit (reduction of mean parasite prevalence) in the higher-prevalence areas, although this benefit decreases as the prevalence level in the population decreases.

### From MTAT to MDA

Simulation results suggest that the most important determinant of success in reducing prevalence is campaign coverage (Fig. 5; Table 3), with a greater effect in areas of higher all-age parasite prevalence. However, in these simulated scenarios, even when achieving high coverage with MDA in areas with a pre-intervention all-age parasite prevalence of less than 10 %, complete elimination will require more than 1 year of campaign implementation. Indeed, preliminary findings from the Southern province MDA intervention trials suggest these strategies are able to reduce parasite prevalence in areas of 8–0.5 %; not elimination, but close enough to warrant moving on to surveillance-based interventions.

### Changing drug

**Fig. 6:**
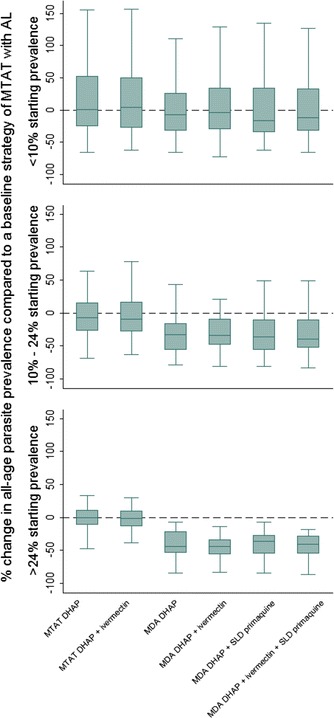
Results of changing drug and testing protocol for anti-malarial drug distribution. Change (%) in the simulated mean parasite prevalence compared to the simulated mean parasite prevalence measured 1 year after implementation of a baseline strategy, for different combinations and testing protocols of DHAP, SLD primaquine, and ivermectin. *Rows* represent different coverage levels achieved by the simulated intervention. For drug combinations at 40 % coverage per round, the baseline includes the existing intervention strategy of three rounds of mass test and treat with AL covering 40 % per round of all ages in the population with 60 days between treatment rounds. For drug combinations at 72 % coverage per round, the baseline includes three rounds of mass test and treat with AL covering 72 % per round of all ages in the population with 60 days between treatment rounds. *Rows* represent results stratified by the pre-intervention mean parasite prevalence in the population as measured by RDT. *Centre solid lines* represent the simulated median value, *green boxes* represent the inter-quartile range, and *capped bars* represent the *upper* and* lower* adjacent values for simulated results using an ensemble of 14 model variants and three random seeds

Changing the intervention drug to DHAP did not result in a simulated reduction in mean parasite prevalence when compared to simulated results using AL. When compared to implementation with AL, adding either SLD primaquine or ivermectin to DHAP did not result in further simulated reduction of mean parasite prevalence 1 year after intervention implementation in either the MTAT or MDA delivery strategies (Fig. 6; Table 4).

**Table 4:** Results of changing drug and testing protocol for anti-malarial drug distribution compared to a strategy of three rounds mass test and treat with artemether-lumefantrine at 72 % coverage per round of all ages of the population with 60 days between rounds

% Change in median parasite prevalence (IQR) compared to baseline strategy, by pre-intervention mean parasite prevalence							
%	Baseline intervention strategy	MTAT with DHAP	MTAT with DHAP + ivermectin	MDA with DHAP	MDA with DHAP + ivermectin	MDA with DHAP + SLD primaquine	MDA with DHAP + ivermectin + SLD primaquine
<10	*MTAT with AL,* 72 % coverage, 60 day frequency between rounds, targeting all ages	0.56 (−25.5, 52.4)	4.5 (−28.0, 50.10	−7.6 (−32.2, 25.5)	−3.6 (−30.5, 33.9)	−17.1 (−35.0, 33.6)	−12.3 (−32.8, 32.2)
10–24		−7.2 (−27.0, 15.3)	−8.8 (−28.6, 16.3)	−*33.0* (−*56.0,* −*16.0*)	−*33.5* (−*48.1,* −*9.5*)	−*35.7* (−*56.7,* −*10.8*)	−*39.0* (−*53.5,* −*10.8*)
>24		−0.2 (−11.9, 11.4)	−1.1 (−13.6, 9.7)	−*44.1* (−*53.6,* −*21.9*)	−*43.6* (−*56.1,* −*33.7*)	−*36.4* (−*55.4,* −*27.2*)	−*40.4* (−*55.1,* −*28.4*)

Italics indicate results that perform better than the baseline strategy

### Sensitivity analysis

**Fig. 7:**
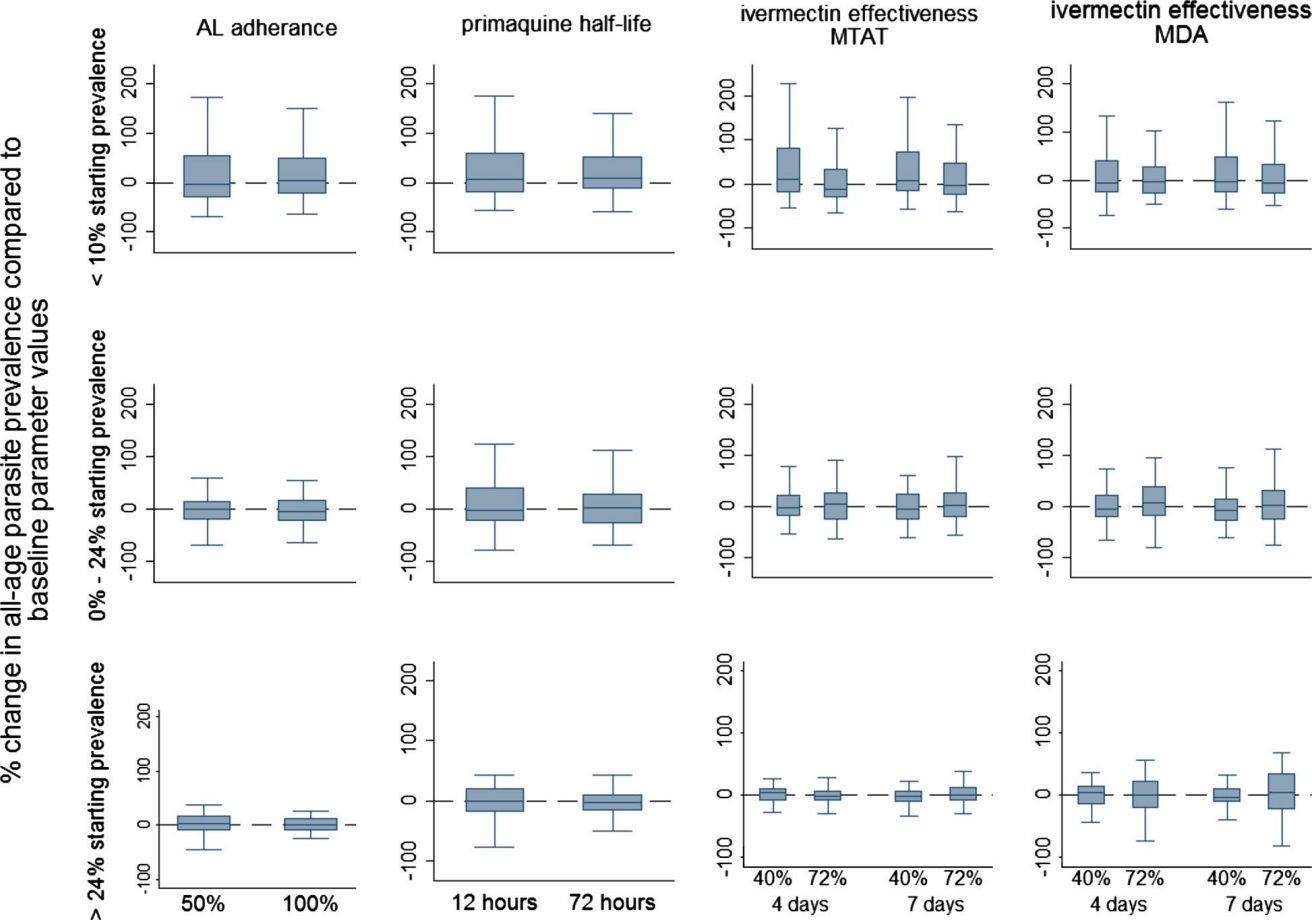
Sensitivity analysis: artemether-lumefantrine (AL) adherence, single low dose primaquine half-life, and ivermectin duration of effectiveness. Change (%) in the simulated mean parasite prevalence compared to the simulated mean parasite prevalence after 1 year of implementation of a baseline strategy, as a result of varying the operational strategy of the intervention. Categories represented in the *columns* include proportion of the population adhering to the artemether-lumefantrine (AL) regimen, the half-life of single low dose primaquine, and duration of effectiveness at different coverage levels for testing strategies of ivermectin paired with AL. The *rows* represent results stratified by the pre-intervention mean parasite prevalence in the population as measured by RDT. For all categories, the baseline includes the existing intervention strategy of three rounds of mass test and treat with AL covering 72 % per round of all ages in the population with 60 days between treatment rounds. Baseline AL adherence is 75 %, baseline primaquine half-life is 48 h, and baseline ivermectin duration of effectiveness is 2 days. *Centre solid lines* represent the simulated median value, *blue boxes* represent the inter-quartile range, and *capped bars* represent the upper and lower adjacent values for simulated results using an ensemble of 14 model variants and three random seeds

Altering the proportion of the population adhering to the drug regimen, altering the half-life of PQ, and altering the duration of effectiveness of ivermectin did not contribute greatly to the overall uncertainty in model predictions (Fig. 7). The range seen in simulation results are therefore more likely to be due to stochasticity and model uncertainty rather than variations in drug half-life/duration of effectiveness or adherence.

## Discussion

In an era where vector control strategies have been able to greatly reduce disease burden across the malaria endemic world, many countries are now evaluating how to transition programmes from control to elimination. Mass distribution of anti-malarials, with or without a diagnostic, are under consideration in many settings to markedly reduce the human parasite reservoir to allow a shift to case detection via surveillance as an intervention. Results of this study are consistent with both modelling and empirical evidence that has shown implementation of drug-based strategies to be effective within a transmission season in settings where sufficient coverage of the population can be achieved [36]. Yet, recent studies on screen and treat for strategies targeting the whole community [37] and specific risk groups [38] have been unable to show sustained impact. This study examined how the OpenMalaria platform, parameterized according to the malaria transmissions dynamics in southern Zambia, can assist the Zambia NMCC to increase the effectiveness of these planned interventions through changes in implementation design.

Results suggest the optimal implementation strategy for deployment of community-based anti-malarial treatments requires a consistent approach addressing the variation in baseline malaria prevalence, rate of imported infections, and the achievement of high coverage in the population. Simulations conducted for this study suggest that an area such as Southern province with heterogeneous transmission and an uncertain infection importation rate, drug-based interventions based on detecting infections using current RDTs will not be sufficient to interrupt transmission. Indeed, as observed in the field study, there was no difference detected at follow-up in parasite prevalence between MTAT intervention and control in either high or low transmission areas [3]. In this setting, a high proportion of low-density infections will be missed by RDTs that would be treated and cleared with MDA.

Simulation results suggest the most important determinant of success in reducing prevalence is the coverage of the population achieved in the campaign. The Southern province field study was designed to show how much impact would be achieved after 1 year of campaign implementation in order to move towards surveillance as an intervention by focal MDA (fMDA) and case investigation. However, even with high coverage with MDA in areas with a pre-intervention all-age parasite prevalence of less than 10 %, simulations suggest that elimination will require more than 1 year of campaign implementation. The inclusion of SLD primaquine (a gametocytocide), and ivermectin (an endectocide), to the drug regimen did not further reduce parasite prevalence within this 1 year post-campaign. It is important to note that mean parasite prevalence measured at earlier time points will yield different results, as the greatest effects will be seen several months post-campaign. Simulation of ACT regimens in a generic setting have been investigated elsewhere, both with and without the option of including SLD primaquine and/or ivermectin [39, 40]. The simulation results presented here are less optimistic about the additional added benefit of PQ and ivermectin in interrupting transmission, although, importantly, the results are evaluated at different time points.

Success of anti-malarial drug administration campaigns depends on the sustained high coverage of vector control interventions. Zambia has been able to achieve high rates of household LLIN ownership and has steadily increased its within-household availability of LLINs through successive mass distribution campaigns. However, LLINs must be used or at least deployed in the household for their full impact to be attributable. Improving simulation estimates by including more direct measures of full coverage and usage of LLINs may yield greater overall reductions in predicted parasite prevalence in combination with the anti-malarial campaigns. Similarly with IRS, improved strategies to target the application of insecticides could be evaluated for specific benefits. Ensuring that transmitting mosquitoes are susceptible to IRS chemicals, that IRS is prioritized to transmission areas, and that full coverage of targeted areas is achieved will maximize the contribution of this intervention where drug strategies are attempted.

### Planning for elimination scenarios: limitations and considerations

In the process of this simulation, we have identified key aspects of both the intervention tools and their delivery; improved data from the field may help in the future to improve the model and its ability to predict outcomes. As noted above, intervention coverage is a critical variable for these population-wide treatment efforts and intervention programming should improve on the quality of the coverage data. For example, most coverage estimates only examine the coverage of the household members in households that were reached; a more accurate estimation of the population denominator and coverage could perhaps be achieved by comparing satellite imaging of houses to actual field geo-position data of which houses were reached by a campaign.

Analysis was conducted with the end-point measurement of reduction in parasite prevalence. This is, indeed, an easily-measured quantity in the field. However, future applications of the OpenMalaria model in the study area should also include an end-point measurement that corresponds to potential vector control interventions, including ivermectin. The goal of ivermectin is not to reduce prevalence, but to reduce transmission. Further analysis measuring reduction in EIR or probability of interrupting transmission would be a more useful way of evaluating the impact of this intervention. In addition to the clear need for direct application of field-measurable quantities, it would be helpful to have a standard definition for defining interruption of transmission for model outputs that is operationally relevant to programme settings. Ideally this would include identifying the number of cases per week or per month in a given health facility catchment area that the health system would be able to handle.

The model has numerous limitations that are inherent in the introduction of many assumptions required for model parameterization. For example, the modelled analysis included a sensitivity analysis for different levels of adherence to AL with the assumption that the majority of non-adherers missed the final dose in the six-dose regimen, with results showing little impact on overall effectiveness. However, non-adherence would have a far greater impact on the cure rate of DHAP if a single dose of this three-dose regimen is missed [41], something not included in this experiment.

The field implementation targeted health facility catchment areas for complete campaign coverage, while the simulation coverage parameterization and analysis is applied to the general population without explicit geographical limits. While model parameterization assumes an individual has the same independent likelihood of receiving the intervention, there are some groups that have a higher risk of being infected. These groups, including adults aged 25–49 that are more highly mobile than the rest of the population, were targeted in the simulation study design by varying the age groups targeted by the campaign. However, because these groups may be less likely to be reached during a general campaign, this higher risk is not captured in the baseline scenario of the model and may affect the impact of the simulated interventions even if a certain level of coverage is reached for the population as a whole.

The model parameterization assumes a conservative value for AL and DHAP prophylaxis based on modelling pharmacokinetics [41]. However, a recent publication of post-treatment prophylaxis of anti-malarials suggests both a shorter duration of protection and a greater difference between the duration of protection between AL and DHAP in people over 5 years of age [42]. This greater difference in prophylaxis benefit would affect the simulation results by increasing the additional transmission prevented by DHAP as compared to AL.

For the ivermectin analysis, the model assumes a common effect of ivermectin no matter the age of mosquito post-emergence, which has been shown to be different in trial settings [14]. Several studies [15] have found a higher mortality rate in mosquitoes exposed to ivermectin than the 85 % assumed in this experiment; this could indicate the experiment design underestimates the effect.

The model assumes a constant rate of yearly case importation (20 cases per 1000 individuals); this rate will undoubtedly vary by season and from location to location even within southern province, and data is not currently available to be able to parameterize this value correctly. Because the OpenMalaria simulations assumed an ongoing rate of imported infections, it is not straightforward from model outputs to distinguish between cases arising from imported infections and cases that were a result of local transmission. This parameter is likely a key driver of the differences in the simulated versus observed results of the model validation shown in Fig. 3. If possible, future iterations of field work could improve the ascertainment of mobility and likely imported versus locally transmitted cases. This includes methods such as parasite genotyping or using human movement via mobile phone data as a proxy of parasite flow [43]. Understanding these dynamics will aid not only in developing more relevant baseline model parameterization, but understanding thresholds for importation in a population on the effect of intervention mixes. Combined with transmission estimates, these metrics can provide a more granular view of what intervention mix to deploy, and where.

Demonstrating elimination, and evaluating the ability of interventions to interrupt transmission, requires metrics that are practical in operational settings. The reproductive number under control settings, *R*_*c*_, has been proposed as a metric to determine whether an area has achieved elimination [44] by calculating the ratio of local cases to imported infections. However, the operational scale at which this should be applied is unclear, as is the geographical definition of infection importation in this context. For example, should importation from higher-transmission zones in the same province be considered as part of the definition, or only importation from a neighbouring country? The area in Southern province currently evaluating anti-malarial administration strategies is bound by the vast Lake Kariba to the east and south, and much lower malaria transmission areas to the north and west, making it an ideal setting to test elimination strategies. A country level metric is likely unsuitable for a country like Zambia which has highly heterogeneous endemicity in different parts of the country. On the other hand, applying such a metric at a more granular administrative level would not confer recognition of elimination by the WHO. Country-specific elimination strategic plans should address how progress will be tracked at both national and sub-national levels.

Other simulation studies and study data suggest the proportion of sub-patent infections missed by RDTs is higher in areas of lower transmission [37, 45, 46], consistent with the simulation results from this study (Fig. 4). Transmission due to asymptomatic infections becomes increasingly important (in relative terms) as cases in a country become fewer and national control programmes approach elimination and prevention of reintroduction. As such, further validation and calibration of models to the prevalence of sub-patent transmissible infections in different epidemiological settings needs to be conducted as new study data becomes available.

Finally, an important limitation for interpreting results is the non-spatially explicit structure of the OpenMalaria model. Focal test and treat and focal MDA strategies that involve targeting specific households, geographic areas or risk groups, and reactive case detection are all implementation strategies in the study area but are not evaluated in the context of this experiment. In addition, there are options for anti-malarial drug administration intervention design that will be relevant in the Southern province that are not explored in this study. Future analyses could examine the potential impact of this larger package of intervention strategies as they are implemented in the same or adjacent geographic areas; such analyses could further contribute to the future programme design by the NMCC.

## Conclusion

The optimal strategy for implementation of community-based interventions involving anti-malarial distribution will vary by background level of malaria prevalence and by rate of infections imported to the area. The ability to achieve high coverage of the population is critical to the success of anti-malarial distribution campaigns. This is reflected in the simulation results showing MDA to be more effective than MTAT at reducing mean parasite prevalence 1 year after intervention implementation. While there was a simulated added benefit of the longer prophylactic effect of DHAP compared to AL, the addition of SLD primaquine and ivermectin, both alone and in combination, did not lead to any appreciable reduction in mean parasite prevalence within the year after implementation. Continued coverage and use of optimized vector control interventions is necessary to maximize the gains in reduction of disease burden. OpenMalaria can be helpful in assisting malaria control and elimination programmes to understand what intervention strategies are most appropriate in different settings, and under which conditions these strategies will be the most effective.
